# Supplementary material for: Giving their all for their offspring: physiological trade-offs in an Andean-Patagonian viviparous lizard in response to global warming
Source: Biol Open. 2025 Sep 22;14(9):bio062159. doi: 10.1242/bio.062159 (PMC12505269; doi:10.1242/bio.062159)
Supplement: Supplementary information [file biolopen-14-062159-s1.pdf]

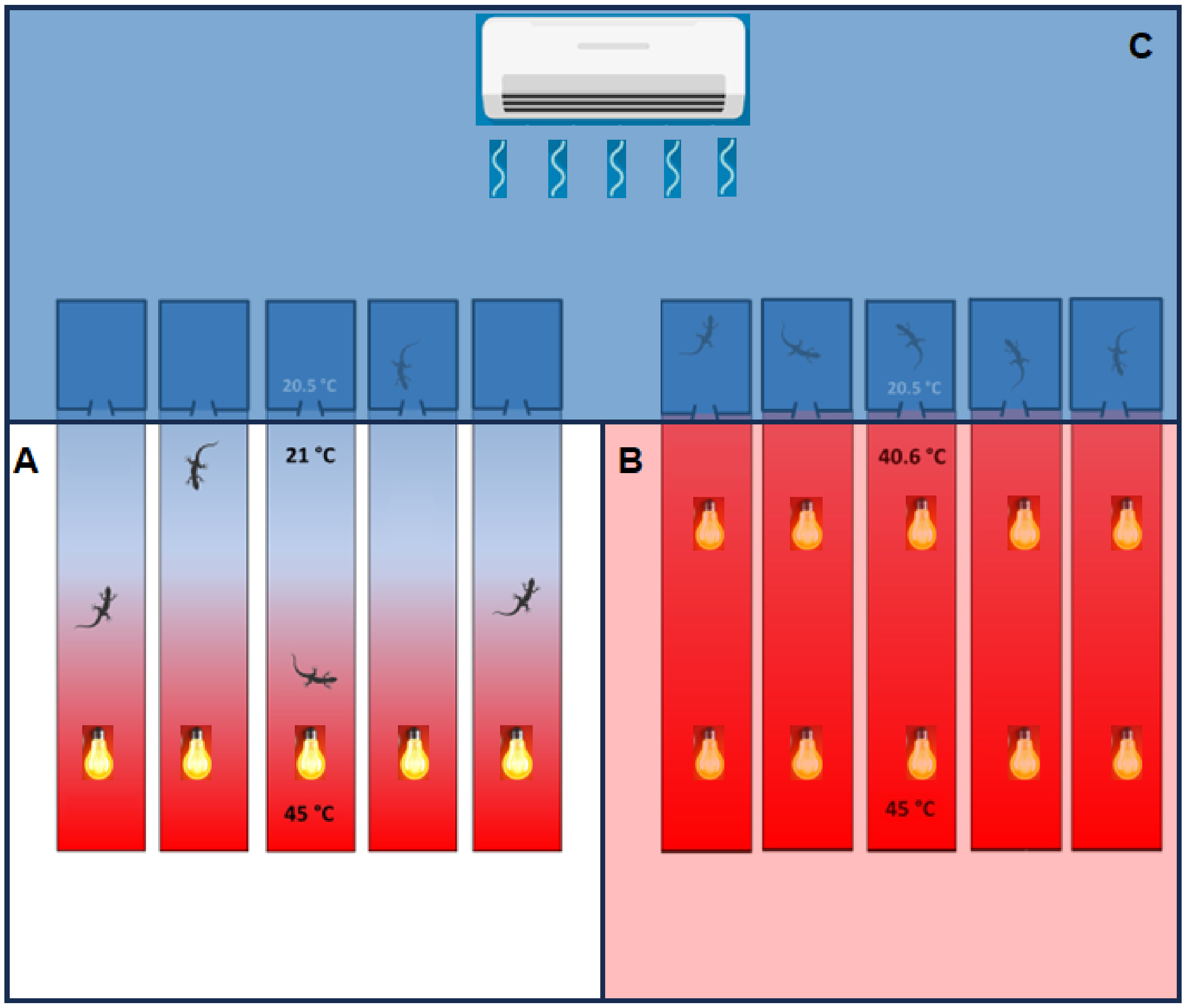

Fig. S1. Representative diagram of the temperature treatments and their thermal surroundings. A) No restriction group (NRG): free access to a thermoregulation area with a thermal gradient from 21 to 45°C (available during activity hours from 9 AM to 6 PM), generated by incandescent lamps (75 W) located 20 cm above one end of the terrarium, and free access to shelter (20.5°C); *n*=14. B) Restriction Group (RG): Same as NRG, but during hours of restriction (4.5 h, between 12 PM and 4:30 PM), individuals could only choose between exposure to 40.6-45°C, achieved by an additional 75 W incandescent lamp positioned 20 cm above and near the opposite end of the terrarium, or refuge at 20.5°C; *n*=12. C) Shelter area: each terrarium contained a shelter with insulated walls (expanded polystyrene) and thermally cooled surroundings, implemented with a room air conditioner, to maintain shelter temperature at 20.5°C. Bold black lines indicate divisions between treatments made with nylon (polyamide) to insulate the thermal conditions of each treatment's surroundings.

Biology Open • Supplementary information

Table S1. Comparative analysis between locomotor speed (V_{max}) of newborns from the No Restriction Group (NRG) and the Restriction Group (RG) in sprint (SR) and long (LR) runs at 21 and 34°C, at birth and 10 days after.

At birth					10 days after birth				
Variable 1	Variable 2	Mean or Median V_{max} (m/s; *n*)	Mean or Median V_{max} (m/s; *n*)	Statistical test (Mann-Whitney or *t*-test; *P*)	Variable 1	Variable 2	Mean or Median V_{max} (m/s; *n*)	Mean or Median V_{max} (m/s; *n*)	Statistical test (Mann-Whitney or *t*-test; *P*)
SR	21°C	RG=0.018, 0.014-0.029 (26)	NRG=0.021, 0.014-0.035 (40)	*U*=474.000; 0.546	SR	21°C	RG=0.016, 0.0125-0.023 (26)	NRG=0.020, 0.013-0.026 (38)	*U*=389.000; 0.151
	34°C	RG=0.030, 0.020-0,037 (26)	NRG=0.026, 0,019-0,035 (40)	*U*=461.000; 0.439		34°C	RG=0.033, 0.033-0,025 (25)	NRG=0.032, 0.024-0,036 (39)	*U*=433.500; 0.457
LR	21°C	RG=0.021±0.007 (26)	NRG=0.019±0.008 (40)	t_{64}=-1.02; 0.311	LR	21°C	RG=0.020, 0,015-0,023 (26)	NRG=0.019, 0,014-0,026 (38)	*U*=485.000; 0.902
	34°C	RG=0.030, 0.027-0.035 (26)	NRG=0.030, 0.021-0.036 (40)	*U*=494.000; 0.733		34°C	RG=0.035±0.006 (25)	NRG=0.031±0.009 (39)	t_{62}=-1.832; 0.072

Values are means±s.e.m or median (max-min) resulting from the comparative analysis between morphometric variables of No Restriction Group (NRG) and Restriction Group (RG). *n*, sample size; *U*, *U-statistic; t*, *t*-statistic with degrees of freedom; *P*, *P*-value (α=0.05).

Biology Open • Supplementary information

Table S2. Comparative analysis within the newborns' No Restriction Group (NRG) and the newborns' Restriction Group (RG) between locomotor speed (V_{max}) in sprint (SR) and long (LR) runs at 21 and 34°C, at birth and 10 days after.

No Restriction Group					Restriction Group				
Variable 1	Variable 2	Mean or Median V_{max} (m/s; *n*)	Mean or Median V_{max} (m/s; *n*)	Statistical test (Wilcoxon or Paired *t*-test; *P*)	Variable 1	Variable 2	Mean or Median V_{max} (m/s; *n*)	Mean or Median V_{max} (m/s; *n*)	Statistical test (Wilcoxon or Paired *t*-test; *P*)
At birth	21°C	SR=0.021, 0.014-0.035 (40)	LR=0.019, 0.014-0.024 (40)	*W*=-344.000; 0.021*	At birth	21°C	SR =0.018, 0.014-0.029 (26)	LR=0.020, 0.016-0.025 (26)	*W*=-17.000; 0.839
	34°C	SR =0.026, 0.019-0.035 (40)	LR=0.030, 0.021-0.036 (40)	*W*=146.000; 0.330		34°C	SR =0.030, 0.020-0.037 (26)	LR=0.030, 0.027-0.035 (26)	*W*=33.000; 0.684
10 days after	21°C	SR =0.020, 0.013-0.026 (38)	LR=0.019, 0.014-0.026 (38)	*W*=-21.000; 0.885	10 days after	21°C	SR =0.018 ±0.002 (26)	LR=0.020 ±0.001 (26)	t_{25}=-2.26; 0.033*
	34°C	SR=0.032, 0.024-0.036 (39)	LR=0.031, 0.027-0.037 (39)	*W*=34.000; 0.818		34°C	SR =0.034 ±0.002 (26)	LR=0.035 ±0.001 (26)	t_{24}=-0.36; 0.720
SR	21°C	Birth=0.021, 0.014-0.035 (40)	10days=0.020, 0.013-0.026 (38)	*W*=-29.000; 0.833	SR	21°C	Birth=0.018, 0.014-0.023 (26)	10days=0.016, 0.012-0.023 (26)	*W*=-163.000; 0.040*
	34°C	Birth=0.026, 0.019-0.035 (40)	10days=0.032, 0.024-0.036 (39)	*W*=191.000; 0.168		34°C	Birth=0.031 ±0.003 (26)	10days=0.034 ±0.002 (26)	t_{24}=-0.635; 0.531
LR	21°C	Birth=0.019 ±0.001 (40)	10days=0.021 ±0.001 (38)	t_{36}=-1.505; 0.141	LR	21°C	Birth=0.021 ±0.001 (26)	10days=0.020 ±0.001 (26)	t_{25}=0.813; 0.424
	34°C	Birth=0.030, 0.021-0.036 (40)	10days=0.031, 0.027-0.037 (39)	*W*=81.000; 0.562		34°C	Birth=0.031 ±0.001 (26)	10days=0.035±0.001(26)	t_{24}=-2.05; 0.051
At birth	SR	21°C=0.024 ±0.002 (40)	34°C=0.029 ±0.002 (40)	t_{39}=-2.52; 0.016*	At birth	SR	21°C=0.023 ±0.002 (26)	34°C=0.031 ±0.003 (26)	t_{25}=-2.10; 0.046*
	LR	21°C=0.019, 0.014-0.024 (40)	34°C=0.030, 0.021-0.036 (40)	*W*=764.000; <0.001*		LR	21°C=0.020, 0.016-0.025 (26)	34°C=0.030, 0.027-0.035 (26)	*W*=291.000; <0.001*
10 days after	SR	21°C=0.020, 0.013-0.026 (38)	34°C=0.032, 0.024-0.036 (39)	*W*=525.000; <0.001*	10 days after	SR	21°C=0.018±0.002(26)	34°C=0.034±0.001(26)	t_{24}=-7.77; <0.001*
	LR	21°C=0.021 ±0.001(38)	34°C=0.031±0.001(39)	t_{37}=-7.49; <0.001*		LR	21°C=0.020 ±0.001 (26)	34°C=0.035 ±0.001 (26)	t_{24}=-9.89; <0.001*

Values are means±s.e.m or median (max-min) resulting from the comparative analysis between sprint (SR) and long (LR) runs, or between 21 and 34°C, or between birth and 10 days after. *n*, sample size; *W*, *W-statistic; t*, *t*-statistic with degrees of freedom; *P*, *P*-value (α=0.05). Asterisks indicate a significant difference (*P<0.05).
